# Responsive Polyesters with Alkene and Carboxylic Acid Side-Groups for Tissue Engineering Applications

**DOI:** 10.3390/polym13101636

**Published:** 2021-05-18

**Authors:** Stella Afroditi Mountaki, Maria Kaliva, Konstantinos Loukelis, Maria Chatzinikolaidou, Maria Vamvakaki

**Affiliations:** 1 Institute of Electronic Structure and Laser, Foundation for Research and Technology-Hellas, Vassilika Vouton, 700 13 Heraklion, Greece; smountaki@gmail.com (S.A.M.); kalivm@iesl.forth.gr (M.K.); loukelisk@yahoo.com (K.L.); mchatzin@materials.uoc.gr (M.C.); 2 Department of Chemistry, University of Crete, Vassilika Vouton, 700 13 Heraklion, Greece; 3 Department of Materials Science and Technology, University of Crete, Vassilika Vouton, 700 13 Heraklion, Greece

**Keywords:** aliphatic polyesters, responsive materials, biodegradable polymers, pH-sensitive polymers, smart polymers, biocompatibility, tissue engineering

## Abstract

Main chain polyesters have been extensively used in the biomedical field. Despite their many advantages, including biocompatibility, biodegradability, and others, these materials are rather inert and lack specific functionalities which will endow them with additional biological and responsive properties. In this work, novel pH-responsive main chain polyesters have been prepared by a conventional condensation polymerization of a vinyl functionalized diol with a diacid chloride, followed by a photo-induced thiol-ene click reaction to attach functional carboxylic acid side-groups along the polymer chains. Two different mercaptocarboxylic acids were employed, allowing to vary the alkyl chain length of the polymer pendant groups. Moreover, the degree of modification, and as a result, the carboxylic acid content of the polymers, was easily tuned by varying the irradiation time during the click reaction. Both these parameters, were shown to strongly influence the responsive behavior of the polyesters, which presented adjustable p*Kα* values and water solubilities. Finally, the difunctional polyesters bearing the alkene and carboxylic acid functionalities enabled the preparation of cross-linked polyester films by chemically linking the pendant vinyl bonds on the polymer side groups. The biocompatibility of the cross-linked polymers films was assessed in L929 fibroblast cultures and showed that the cell viability, proliferation, and attachment were greatly promoted on the polyester surface, bearing the shorter alkyl chain length side groups and the higher fraction of carboxylic acid functionalities.

## 1. Introduction

Aliphatic polyesters such as polylactide (PLA), polycaprolactone (PCL), and polyglycolide have been extensively investigated as promising biodegradable biomaterials in drug delivery and tissue engineering applications due to their biocompatibility with a variety of cells and tissues [1,2,3]. Besides these notable properties, polyesters also present several drawbacks, among which, the most important are their hydrophobicity and the absence of functional pendant groups along the polymer backbone. These groups are particularly attractive because they allow to tune the physicochemical and biological properties of the biomaterials at will. Amine, hydroxyl, and carboxylic acid side groups have been shown to influence the adhesion, proliferation, and differentiation of cells on biomaterial scaffolds [4]. Moreover, they can be utilized to conjugate bioactive molecules onto the polymer, including proteins and peptides, that regulate and guide the cellular activity. Finally, the introduction of functional groups that are sensitive to external stimuli, such as the solution pH and temperature, light irradiation, and others, can impart exemptional properties to the polymers, rendering them “smart” materials. It is evident from the above that the presence of functional pendant groups along the polyester backbone is highly desirable and can lead to novel materials with diverse, multi-functional properties that can have great potential.

The synthesis of biodegradable aliphatic polyesters bearing functional pendant groups has received increased attention during the last few years. Several synthetic approaches have been developed for the synthesis of such materials, including the ring-opening polymerization of functional lactones [5,6,7,8], lactides [6,9], and O-carboxyanhydrides [10,11]. However, these methods involve either a complex multistep synthetic route or a reaction performed under very strict conditions. Recently, the synthesis of functional polyesters via the radical ring-opening copolymerization of cyclic ketene acetals with conventional vinyl monomers was reported [12,13]. Furthermore, polyesters with reactive side groups have been synthesized by scalable synthetic methods, i.e., the step-growth polymerization of difunctional comonomers. The latter approach has been used to prepare polyesters bearing diverse functional moieties such as azide [14], hydroxyl [15], alkene [16], alkyne [17], and phosphate [18] groups. Another approach involved the reaction of telechelic polyester diols with “chain extenders”, such as small functional diols and diisocyanates to introduce functional groups along the polyurethane backbone [19].

Despite the significant progress in the field over the last years, the synthesis of functional polyesters and their investigation for use in tissue engineering applications is scarce in the literature. In particular, nanofibrous scaffolds based on a hydroxyl-functionalized polyester, poly[hydroxymethylglycolide-*co*-(ε-caprolactone)], were loaded with the vascular endothelial growth factor (VEGF) and were shown to support the growth of HUVECs in vitro [20]. More recently, electrospun scaffolds were fabricated using a poly(glycerol sebacate)/PLLA mixture and were shown to support cardiomyocytes in vitro and to induce neovascularization upon implantation in a mouse heart [21]. In another study, elastic scaffolds comprising poly(octamethylene maleate (anhydride) 1,2,4-butanetricarboxylate), which bears carboxylic acid groups, were proven to support rat cardiac cell adhesion in vitro and presented an acute in vivo host response, which was comparable to poly(L-lactide) [22]. Three-dimensional scaffolds based on poly(sebacoyl diglyceride) phosphate, bearing both hydroxyl and phosphate side moieties, have been demonstrated to promote the adhesion and proliferation of osteoblasts [18]. In a related study, functional polyesters with alkene and alkyne side groups were 3D-printed into scaffolds, followed by conjugation of azide-heparin, and were shown to enhance the osteogenic differentiation of human mesenchymal stem cells (hMSCs) [17]. Finally, water-responsive shape memory scaffolds based on poly(fumaric acid-*co*-1,7-octadiene diepoxide-*co*-terephthalic acid) promoted the adhesion and proliferation of cardiomyoblast H9C2 cells and were proposed for surgical applications [23]. All the studies have focused on the biological properties of the functional polyesters and compared them to those of the traditional polyesters, such as PLA [22] and poly(lactic-*co*-glycolic acid) (PLGA) [21]. However, none of these studies have investigated the pH-responsive character of the functional polyesters as well as their potential in the development of cross-linked hydrogels for tissue regeneration.

Motivated by the great interest in polyesters as biodegradable biomaterials for use in tissue engineering applications and the recent advances in the development of novel functional polyesters, we report in this study the synthesis of “smart”, pH-responsive biodegradable polyesters bearing alkene/carboxylic acid side groups and their potential for use in tissue engineering applications. The presence of the carboxylic acid side groups provides pH-responsive properties to the polymers and render them water soluble, whereas the alkene groups can be further utilized for the formation of stable hydrogels and cross-linked polymer films. For the preparation of the functional polymers, first a polyester with alkene side groups was synthesized by condensation copolymerization of commercially available difunctional monomers, trimethylolpropane allyl ether (TPAE) and adipoyl chloride (ADC), at room temperature in the absence of any metal catalyst [16]. The presence of alkene side groups offers great advantages since they can be easily modified via appropriate post-polymerization chemistries such as the thiol-ene click reaction. Thiol-ene click chemistry has been employed in polymer synthesis and modification, bioconjugation, labelling, surface functionalization, as well as hydrogel formation [24,25,26,27]. It comprises an attractive approach for the functionalization of biodegradable polyesters because it can be performed at room temperature under mild irradiation for several minutes up to a few hours, while avoiding possible main chain degradation of the polyester backbone. The synthesized poly(trimethylolpropane allyl ether-*alt*-adipoyl ester) P(TPAE-*alt*-AD) copolymer containing alkene side groups was modified to bear pH-responsive carboxylic acid moieties by grafting mercaptocarboxylic acids via a photo-induced thiol-ene click reaction. Two mercaptocarboxylic acids with different alkyl chain lengths, 3-mercaptopropionic acid (Prop) and thioglycolic acid (Glyc), were used, while the degree of modification of the alkene side groups of the polymer was adjusted at 50, 80, and 100 mol% by varying the irradiation time during the thiol-ene click reaction. To evaluate the cytocompatibility of the functional polyesters, thin films were prepared by spin coating on pre-silanized glass substrates bearing polymerizable methacrylate groups. The films were cross-linked and covalently attached onto the substrates by a photo-induced free-radical coupling of the pendant alkene moieties. The suitability of the polymer films to serve as culture substrates was investigated by evaluating the cell viability, proliferation, adhesion, and morphology of L929 fibroblasts cultured on them.

## 2. Materials and Methods

### 2.1. Materials

Trimethylolpropane allyl ether (98%), adipoyl chloride (98%), pyridine (99.5%), 3-mercaptopropionic acid (≥99%), thioglycolic acid (98%), 3-(trimethoxysilyl)propyl methacrylate (98%), 2,2-dimethoxy-2-phenyl-acetophenone (99%), chloroform (≥99.8%), methanol (≥99.8%), dimethylformamide (≥99.8%), and Hellmanex III were purchased from Sigma-Aldrich (Steinheim, Germany). Ethanol (≥99.8%) was obtained from Honeywell (Seetze, Germany) and toluene (≥99.8%) from Fisher Scientific (Loughborough, UK). Dichloromethane (analytical grade), n-hexane (96%), and diethyl ether (99.8%) were purchased from Scharlau (Sentmenat, Spain). Dichloromethane was dried over calcium hydride and distilled under a nitrogen atmosphere prior to use. All the other solvents and chemicals were used without further purification.

Trypsin/EDTA (0.25%), phosphate buffer saline (PBS), Amphotericin-B (fungizone), penicillin/streptomycin (P/S), were all purchased from Gibco ThermoFisher Scientific (Waltham, MA, USA), Roswell Park Memorial Institute 1640 medium (RPMI) with stable glutamine and fetal bovine serum (FBS) from PAN-Biotech (Aidenbach, Germany) and PrestoBlue^TM^ reagent for cell viability from Invitrogen Life Technologies (Carlsbad, CA, USA). The L929 fibroblastic cell line was purchased from the German collection of microorganisms and cell cultures (DSMZ GmbH, Braunschweig, Germany).

### 2.2. Synthesis of Poly(TPAE-alt-AD)

The functional polyester with pendant alkene groups was synthesized by a condensation copolymerization of a diol and a diacyl chloride, as described by Yan and coworkers [16]. In a typical procedure, adipoyl chloride (ADC) (8.43 mL, 57.97 mmol) was added dropwise in a solution of trimethylolpropane allyl ether (TPAE) (10 mL, 57.97 mmol) in freshly distilled dichloromethane (DCM) (120 mL), containing dry pyridine (10.31 mL, 127.53 mmol) under a N_2_ atmosphere, in an ice bath. The polymerization was allowed to proceed for 67 h at room temperature. Subsequently, the salt formed was removed by filtration and the filtrate was evaporated using a rotary evaporator (Heidolph Rotary Evaporator, Laborota 4000 efficient, Heidolph Instruments GmbH & Co KG, Schwabach, Germany) to reduce the volume of the solution. The final product was collected by precipitation in cold methanol. ^1^H NMR (500 MHz, CDCl_3_): δ 5.77–5.90 ppm (m, CH_2_=C**H**–), δ 5.12–5.27 ppm (m, C**H_2_**=CH–), δ 4.01 (s, –C(O)OC**H_2_**–), δ 3.90–3.93 ppm (m, –OC**H_2_**CH=CH_2_), δ 3.29 ppm (s, –C**H_2_**OCH_2_–), δ 2.30–2.34 ppm (t, –O(O)CC**H_2_**–), δ 1.61–1.66 ppm (m, –O(O)CCH_2_C**H_2_**–), δ 1.40–1.49 ppm (q, –C**H_2_**CH_3_), δ 0.83–0.88 ppm (t, –CH_2_C**H_3_**).

### 2.3. Synthesis of Functional Polyesters with Carboxylic Acid Side Groups

The polyester bearing alkene side groups, P(TPAE-*alt*-AD), was modified using mercaptocarboxylic acids via a pho-induced thiol-ene reaction, to obtain the carboxylic acid functionalized polyesters. For this, P(TPAE-*alt*-AD) (1.25 g, 4.40 mmol) and dimethylformamide (DMF) (5 mL) were introduced in a Schott–Duran round-bottom flask, while 2,2-dimethoxy-2-phenylacetophenone (DMPA) (0.2 eq., 225.6 mg, 0.880 mmol) was added as the free-radical photoinitiator. The mixture was stirred with 3-mercaptopropionic acid (1.5 eq., 575 μL, 6.60 mmol) or thioglycolic acid (1.5 eq., 458.8 μL, 6.60 mmol) for 15 min, under a N_2_ atmosphere, at room temperature. Next, each solution was irradiated with UV light (λ = 365 nm, Spectroline ENF-280C, Spectronics Corporation, Westbury, NY, USA). Three copolymers, with a degree of modification of the alkene side groups to carboxylic acid moieties of 50%, 80%, and 100%, were prepared by varying the irradiation time. Finally, the reaction mixture was dialysed against methanol for 24 h to remove the unreacted mercaptocarboxylic acid, the photoinitiator, and DMF. The purified product was collected by precipitation in a cold n-hexane/diethyl ether mixture (ratio 50:50). ^1^H NMR (300 MHz, acetone–d6) for PE-Prop100: δ 4.02 ppm (s, –C(O)OC**H_2_**–), δ (3.49–3.52) ppm (t, –OC**H_2_**CH_2_–), δ 3.56 ppm (s, –CC**H_2_**O–), δ 2.73–2.78 ppm (t, HO(O)CC**H_2_**–), δ 2.57–2.65 ppm (q, –C**H_2_**SC**H_2_**–), δ 2.37 ppm (s, –O(O)CC**H_2_**–), δ 1.77–1.86 ppm (m, –OCH_2_C**H_2_**–), δ 1.65 ppm (s, –O(O)CCH_2_C**H_2_**–), δ 1.44–1.51 ppm (q, –C**H_2_**CH_3_), 0.87–0.91 ppm (t, –CH_2_C**H_3_**), and for PE-Glyc100: δ 4.02 ppm (s, –C(O)OC**H_2_**–) δ 3.48–3.52 ppm (t, –OC**H_2_**CH_2_–), δ 3.36 ppm (s, –CC**H_2_**O–), δ 3.25 ppm (s, –(O)CC**H_2_**S–), δ 2.69–2.74 ppm (t, –CH_2_C**H_2_**S–), δ 2.37 ppm (s, –O(O)CC**H_2_**–), δ 1.80–1.89 ppm (m, –OCH_2_C**H_2_**CH_2_S–),), δ 1.64 ppm(s, –O(O)CCH_2_C**H_2_**–), δ 1.43–1.50 ppm (q, –C**H_2_**CH_3_), δ 0.86–0.91 ppm (t, –CH_2_C**H_3_**).

### 2.4. Preparation of Polyester-Based Thin Films

Thin films of the functional polyesters were prepared on glass coverslips (100 μm thickness, 13 mm diameter). First, the glass substrates were sonicated in a Hellmanex aqueous solution for 30 min, followed by thorough rinsing with deionized water and drying under a N_2_ gas stream. Subsequently, the coverslips were immersed in a 5 *v*/*v*% solution of 3-(trimethoxysilyl)propyl methacrylate (MAPTMS) in anhydrous toluene, under a N_2_ atmosphere, at room temperature for 18 h. Next, the modified substrates were extensively washed with toluene and were finally dried with a N_2_ gas stream. Separately in a vial, a 10 *w/v*% solution of the functional polyester in chloroform (CHCl_3_) was prepared, and 1 wt.% of DMPA was added as the photoinitiator. The solution was filtered twice through a PTFE syringe filter (0.45 μm) and 100–200 μL of the filtered solution were spin-coated, using an SPS spin coater (model Spin 150, SPS-Europe, Putten, The Netherlands) (1600 rpm, 120 s, 1000 rpm/s), on the MAPTMS modified glass substrates. The films of the alkene functional polyesters were next cross-linked via their vinyl pendant moieties and were bound to the methacrylate double bonds of the substrate by irradiation with UV light (λ = 365 nm) for 90 min. Finally, the films were dried overnight under vacuum at 40 °C (Gallenkamp OVA 031.XX 1.5, Fistreem International Ltd. Cambridge, UK) and were next rinsed multiple times with ethanol and deionized water before use.

### 2.5. Characterization Techniques

The number average molar mass (*M*_n_’s) and the molecular weight distributions (*M*_w_/*M*_n_’s) of the P(TPAE-*alt*-AD) polyesters, were determined by size exclusion chromatography (SEC). The system comprised a Waters 515 HPLC pump, two PLgel mixed D and mixed E (Agilent Technologies, Santa Clara, CA, USA) columns at 40 °C, and a refractive index (RI) Waters 410 detector (Waters, Milford, MA, USA). THF with 2 *v*/*v*% triethylamine was used as the eluent at a flow rate of 1 mL min^−1^. The calibration curve was based on five narrow PMMA standards with molar mass ranging from 850 to 138,600 g mol^−1^. The synthesized functional polyesters were characterized by proton nuclear magnetic resonance (^1^H NMR) spectroscopy using a Bruker AMX-500 spectrometer (Bruker, Rheinstetten, Germany). Spectra were recorded in CDCl_3_ or acetone-d6 as the deuterated solvents at 25 °C. Attenuated total reflection-Fourier transform infrared (ATR-FTIR) spectra were recorded on a Nicolet 6700 spectrometer (Thermo Fisher Scientific, Waltham, MA, USA). For each spectrum, 128 scans were collected in the 500–4000 cm^−1^ range. The aqueous solution behavior of the synthesized polyesters bearing carboxylic acid side groups was investigated as a function of the solution pH by potentiometric titration measurements. In a typical experiment, first, the polymer was dissolved in water at 1 wt.% and the solution pH was adjusted to pH~12 using 0.1 M NaOH. After stirring for 30 min at RT, the polymer solution was titrated using 20–100 μL aliquots of 0.01 M HCl and the decrease of the solution pH was monitored as a function of the amount of acid added. One minute equilibration time was allowed between each addition of the titrant. The pH was measured with a WTW inoLab pH7110 (Xylem Analytics Germany Sales GmbH & Co. KG, Weilheim, Germany) pH meter and titration curves in the pH range from 12 down to 3 were obtained. The pH-dependent solubility of the carboxylic acid functionalized polyesters was studied by optical transmission measurements at λ = 650 nm using a Perkin Elmer Lambda 25 UV/Vis Spectrophotometer (Perkin Elmer, Llantrisant, UK). For this, a 2 wt.% aqueous polymer solution at pH = 12 was prepared and the pH of the solution was gradually decreased by the addition of 20–100 μL aliquots of 0.1 M HCl. The transmittance of the solution was measured as a function of the solution pH at room temperature. Thermogravimetric Analysis (TGA) was performed using a Perkin Elmer Pyris Diamond TG/DTA instrument (Llantrisant, UK). In a typical measurement, 15 mg of the sample were placed in an aluminum holder and were heated under a constant nitrogen flow, from room temperature up to 500 °C, at a heating rate of 10 °C/min. A UV lamp was used for the preparation of the acid-functionalized polyesters and the thin polyester films on the glass substrates. The thickness of the films was measured using a Veeco Dektak 150 Optical Profilometer (Plainview, NY, USA). The wettability of the polymer films was assessed by static water contact angle (WCA) measurements using a contact angle goniometer (OCA-40, DataPhysics Instruments GmbH, Filderstadt, Germany) and the sessile drop method. A 5 μL droplet of nanopure water was used and the contact angles were calculated from the digital images of the water droplets deposited on the surfaces, recorded by a camera, using the appropriate software. Each measurement was carried out five times (n = 5).

### 2.6. Cell Culture of L929 and Seeding onto the Polyester Films

The biocompatibility assessment was conducted using L929 fibroblast cells of passage 13 and lower (cat. No. ACC-2, DSMZ GmbH, Braunschweig, Germany). The L929 cell line was chosen primarily due to the excellent adaptability that the fibroblasts present to different environments, making them ideal for cytocompatibility testing. Cells were cultured in a humidified incubator at 37 °C and 5% CO_2_ in RPMI 1640 culture medium (w/s stable glutamine), enriched by the addition of 10% fetal bovine serum (FBS), 1% penicillin/streptomycin, and 1% amphotericin B. The cells were trypsinized using a 0.25% trypsin/EDTA solution when they reached their maximum level of confluency.

For each cell viability and adhesion experiment, 2 × 10^4^ cells were seeded onto the modified polyester films spin coated on glass substrates in 24-well plates. The same number of cells was seeded onto tissue-culture-treated polystyrene (TCPS) wells used as a control surface. Prior to the cell seeding, the polyester films were sterilized by exposure to UV light for 15 min and washed once with the culture medium. Then, a 10 μL drop of the cell suspension was added into each well, which was then filled with 500 μL culture medium, and the plate was placed in a humidified cell culture incubator (ThermoFisher Scientific, Roskilde, Denmark). Medium change was carried out every two to three days.

### 2.7. Cell Viability and Proliferation Assay

The PrestoBlue^TM^ cell viability assay is a technique widely used to assess the cytotoxicity of the examined materials. It is a resazurin-based colorimetric method, which allows to indirectly measure the number of living cells, by calculating the level of reduction of resazurin in the environment of the cells [1,3]. In its oxidized form, resazurin has a deep blue color, but when reduced, it becomes purple. The intensity of the purple color is proportional to the reduction potential of the living cells, which in turn allows the quantification of the cell viability by means of a spectrophotometer. The PrestoBlue^TM^ assay was conducted after 2, 4, and 7 days in culture. Following the removal of the culture medium, the cells were incubated for 1 h at 37 °C in a mixture of PrestoBlue^TM^ and fresh medium at a ratio of 1:10 *v*/*v*. Next, 100 μL of the mixed solution from every sample were transferred into a 96-well plate and the absorbance was measured at 570/600 nm in a spectrophotometer (Synergy HTX Multi-Mode Microplate Reader, BioTek, Bad Friedrichshall, Germany). Following the measurements, the culture medium was replaced in each well. Three independent experiments each in triplicates (n = 9) were performed and analyzed to evaluate the cell viability.

### 2.8. Optical Microscopy

The cell seeded polyester films were observed using an inverted optical microscope (Axiovert 200, Carl Zeiss, Berlin, Germany) and the photographs were taken by employing a ProgRes CF scan camera and its compatible software ProgRes Capture Pro (Jenoptik Optical Systems GmbH, Berlin, Germany). In order to evaluate the cell adhesion and morphology, optical microscopy images were obtained for each sample at day 4, as the morphological features of cells can be well recognized at this particular experimental time point due to their moderate number, and were compared to the TCPS control surface.

### 2.9. Statistical Analysis of the Cell Viability

The GraphPad Prism software version 8.0 (GraphPad Software, San Diego, CA, USA) was used to perform the statistical analysis of the cell viability experiments. Specifically, the statistical analysis was conducted using the ANOVA *t*-test method in order to determine the statistical significance of cell viability between the cells seeded on the polyester films and the TCPS control. The analysis was performed for each experimental time point (day 2, 4, and 7). A *p*-value of <0.05 is considered statistically significant and was designated with one asterisk, *p* < 0.01 is depicted with two asterisks, and *p* < 0.001 with three asterisks.

## 3. Results and Discussion

### 3.1. Synthesis and Characterization of the Alkene Functionalized Polyesters

**Scheme 1:**
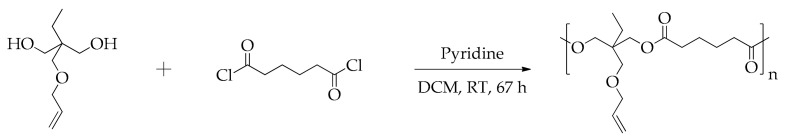
Synthetic procedure followed for the preparation of the P(TPAE-*alt*-AD) polyester.

**Figure 1:**
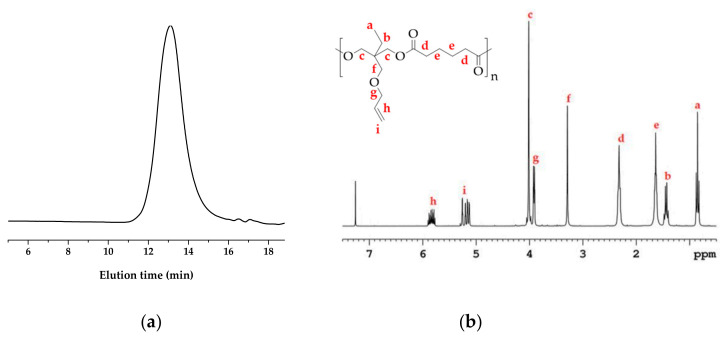
SEC curve (**a**) and ^1^H NMR spectrum (**b**) of the P(TPAE‒*alt*‒AD) polyester, where the peaks labeled (a–i) correspond to the protons of the polyester shown above.

The functional polyesters P(TPAE-*alt*-AD) containing pendant alkene groups were prepared by a polycondensation reaction of trimethylolpropane allyl ether (TPAE), an alkene-functionalized diol, and adipoyl chloride (ADC), a diacyl chloride with six carbon atoms. The polymerization was carried out at room temperature in the absence of any metal catalyst (Scheme 1). After synthesis and purification of the product, by precipitation in methanol, the number average molar mass and the molecular weight distribution of the synthesized polyester were found M_n_ = 33,500 g/mol and M_w_/M_n_ = 1.3, respectively by SEC (Figure 1a). The relatively broad polydispersity of the polymer is expected for a polycondensation reaction. Furthermore, two characteristic peaks at 1.63 ppm (He) and 2.32 ppm (Hd), attributed to the adipoyl monomer repeat units, and two peaks at 5.82 ppm (Hh) and 5.0–5.3 ppm (Hi), assigned to the vinyl group present in the TPAE monomer repeat units, were observed in the ^1^H NMR spectrum of the product, verifying the successful synthesis of the P(TPAE-*alt*-AD) polyester (Figure 1b).

### 3.2. Synthesis and Characterization of the pH-Responsive Polyesters

**Scheme 2:**
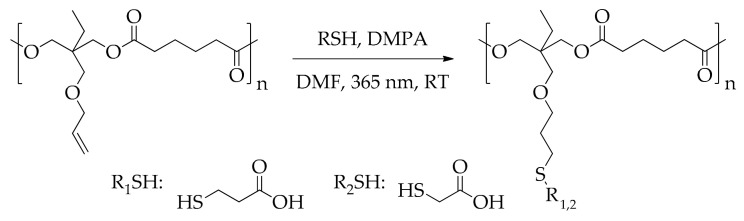
Synthetic procedure followed for the preparation of pH-responsive polyesters containing carboxylic acid side groups of different alkyl chain length.

Next, the alkene-containing polyester was modified to bear carboxylic acid pendant groups via a photo-induced thiol-ene click reaction and produce pH-responsive polyesters. P(TPAE-*alt*-AD) was reacted with either thioglycolic acid (Glyc) or 3-mercaptopropionic acid (Prop), containing two and three carbon atoms, respectively, to obtain two different polyesters bearing carboxylic acid side groups along the polymer backbone (Scheme 2). The different alkyl length of the side groups is expected to influence both the physicochemical and biological properties of the pH-responsive polyesters which are discussed below. The photoinduced thiol-ene click reactions were carried out at room temperature by irradiation with a UV-lamp emitting at 365 nm, using 1.5 eq. of the mercaptocarboxylic acids with respect to the pendant alkene groups of the polyester, in the presence of DMPA as the photoinitiator. Polyesters with 50%, 80%, and 100% carboxylic acid side groups were obtained by varying the irradiation time from 60 s, 180 s, and 5 min for 3-mercaptopropionic acid and 40 s, 60 s, and 4 min for thioglycolic acid, at a constant mercaptocarboxylic acid/alkene mole ratio of 1.5. In the following, the synthesized polyesters obtained by the photo-induced thiol-ene click reaction are named as PE-GlycXX and PE-PropXX, where PE stands for the polyester main chain, Glyc and Prop represent the glycolic acid and propionic acid side groups, and XX is the degree of modification of the alkene groups to the carboxylic acid functionalities. Thus, PE-Prop50, PE-Prop80, and PE-Prop100 represent the propionic acid functionalized polyesters and PE-Glyc50, PE-Glyc80, and PE-Glyc100 denote the glycolic acid functionalized polymers.

**Figure 2:**
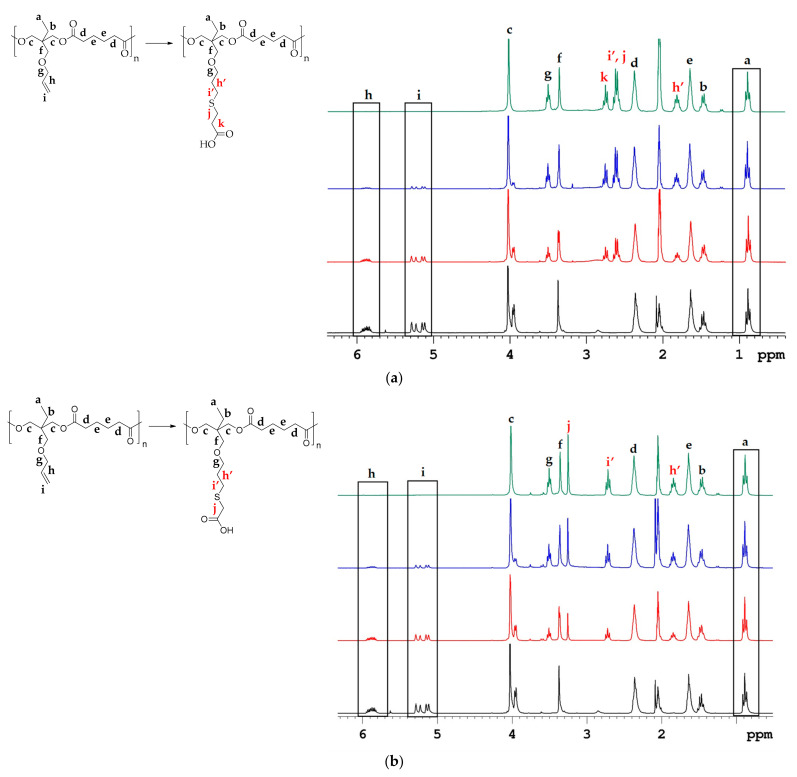
^1^H NMR spectra of (**a**) P(TPAE‒*alt*‒AD) (black line), PE‒Prop50 (red line), PE‒Prop80 (blue line), and PE‒Prop100 (green line) and (**b**) P(TPAE‒*alt*‒AD) (black line), PE‒Glyc50 (red line), PE‒Glyc80 (blue line), and PE‒Glyc100 (green line), in d6-acetone, where the peaks labeled (a–k) correspond to the protons of the repeat units of the polyester shown on the left.

The successful post-synthesis modification of the polyester to bear carboxylic acid pendant groups and the degree of modification of the alkene groups of P(TPAE-*alt*-AD) were investigated by ^1^H NMR spectroscopy. The appearance of three new peaks at 1.82 ppm (Hh’), 2.61 ppm (Hi’, Hj), and 2.75 ppm (Hk), attributed to the newly formed thio-ether groups and the attached propionyl group, verify the successful polymer modification with Prop (Figure 2a). The degree of modification was quantified by ratioing the peak integrals of the methylene protons (Ha) of the polymer backbone, at 0.89 ppm, to the remaining vinyl protons (Hh and Hi), at 5.86 ppm and 5.0–5.3 ppm, respectively. The ratio of the integrals of the peaks verified the degree of modification of the vinyl groups to the carboxylic acid moieties at 49% 83%, and 100% for the three samples. Similar were the results for the polyester modified with Glyc. The ^1^H NMR spectra showed three new peaks at 1.85 ppm (Hh’), 2.72 ppm (Hi’), and 3.25 ppm (Hj), attributed to the newly formed thio-ether groups and the attached glycolic group and verified the successful modification of the polyester (Figure 2b). By ratioing the integrals of the peaks at 5.87 ppm (Hh) and 5.0–5.3 ppm (Hi) to the peak at 0.89 ppm (Ha) a 46%, 83%, and 100% degree of modification of the alkene moieties of the polyester, to the carboxylic acid functionalities, was calculated.

**Figure 3:**
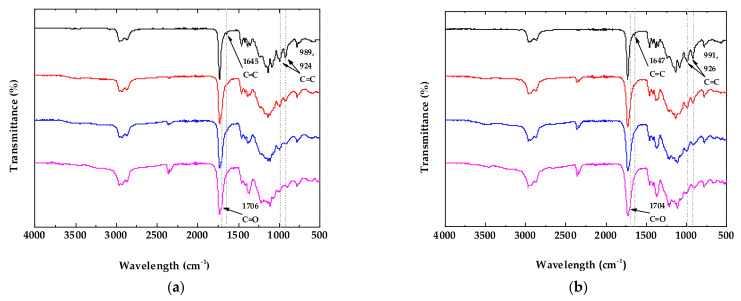
FTIR spectra of (**a**) P(TPAE‒*alt*‒AD) (black line), PE‒Prop50 (red line), PE‒Prop80 (blue line), and PE‒Prop100 (magenta line) and (**b**) P(TPAE‒*alt*‒AD) (black line), PE‒Glyc50 (red line), PE‒Glyc80 (blue line), and PE‒Glyc100 (magenta line).

The functional polyesters were also characterized by ATR-FTIR spectroscopy (Figure 3). The ATR-FTIR spectra of the modified polyesters, bearing the carboxylic acid side groups, show a new peak at 1706 cm^−1^ assigned to the stretching vibration of the C=O bond of the carboxylic acid moieties and a decrease of the stretching vibrations attributed to the C=C bond of the vinyl side groups at ~1645, ~990, ~925 cm^−1^, verifying the successful derivatization of the polymer. The gradual increase of the intensity of the peak attributed to the carbonyl bond of the carboxylic acid groups and the monotonous decrease of the intensity of the peaks due to the carbon–carbon double bond is also evident as the degree of modification of the vinyl groups increases, in agreement with the ^1^H NMR results discussed above.

**Figure 4:**
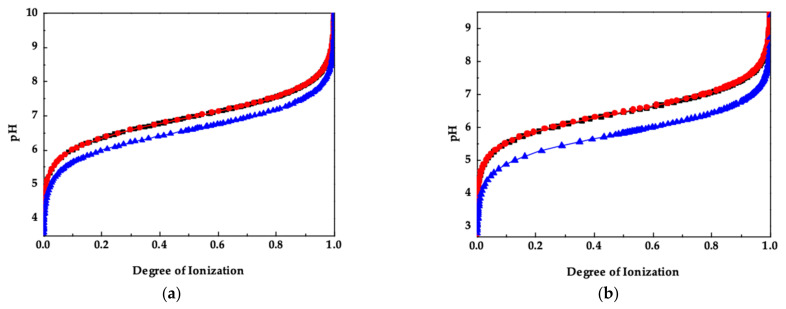
Potentiometric titration data plotted as the pH vs. the degree of ionization (0 < *α* <1) of the carboxylic acid side groups for (**a**) PE‒Prop50 (■), PE‒Prop80 (●) and PE‒Prop100 (▲) and (**b**) PE‒Glyc50 (■), PE‒Glyc80 (●) and PE‒Glyc100 (▲).

**Figure 5:**
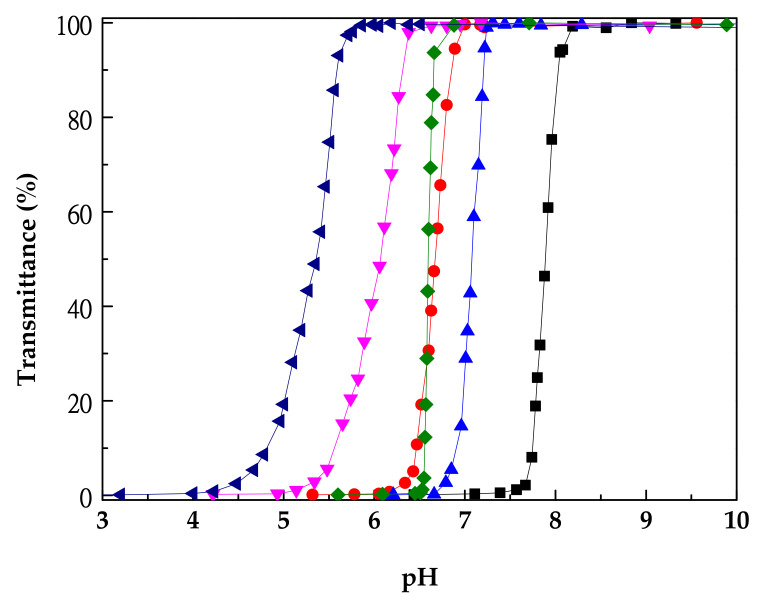
Transmittance of a 1 wt.% aqueous solution of PE‒Prop50 (■), PE‒Prop80 (▲), PE‒Prop100 (♦), PE‒Glyc50 (●), PE‒Glyc80 (▼) and PE‒Glyc100 (◀) as a function of the solution pH.

Next, the aqueous solution behavior of the pH-sensitive polyesters was investigated. Figures S1 and S2 show the potentiometric titration curves for the PE-PropXX and PE-GlycXX polymers, respectively. The titration curves exhibit a plateau region at pH ~ 7 for PE-PropXX and pH ~ 6 for PE-GlycXX. Moreover, the plateau increases with the degree of modification of the vinyl groups of the polymers, suggesting that the carboxylic acid side groups participate in the acid-base equilibrium in this region. From this data, the degree of ionization (*α*) of the polymer, in the range 0 < *α* < 1, was calculated as the net uptake of H^+^ ions by the carboxylate anions, assuming that all H^+^ from the added HCl are taken up by the polymer groups. The pH as a function of the degree of ionization of the carboxylic acid groups of the polymer is shown for PE-PropXX and PE-GlycXX in Figure 4 and Figure 5, respectively, and corresponds to the plateau region of the potentiometric titration curves. As seen in Figure 4, PE-Prop50 and PE-Prop80 are 100% ionized at pH = 9.0 and 0% ionized at pH = 4.8, whereas PE-Prop100 is fully ionized at pH = 8.6 and fully protonated at pH = 4.4. Similarly, Figure 5 shows that PE-Glyc50 and PE-Glyc80 are 100% ionized at pH = 8.5 and 0% ionized at pH = 4.2, while PE-Glyc100 is fully ionized at pH = 7.8 and fully protonated at pH = 3.6. The p*K*α is defined as the pH at 50% ionization of the weak acid groups and corresponds to *α* = 0.5. The effective p*K*α value of the acidic groups for PE-Prop100 was found at 6.6 and increased to 6.9 for PE-Prop50 and PE-Prop80 (Figure 4 and Table 1). These values are significantly higher than the p*Kα* of 3-mercaptopropionic acid at 4.3. The difference is attributed to both the polyelectrolyte effect and the hydrophobicity of the polymer, which results in a reduction of the dielectric constant and a strengthening of the Coulombic interactions, thus inhibiting the ionization of the carboxylic acid groups of the polymers. The presence of non-modified alkene side groups for PE-Prop50 and PE-Prop80 increased even further the hydrophobic nature of the copolymer and therefore led to higher effective p*K*α values.

Similar results were obtained for the glycolic acid functionalized polyesters. The effective p*K*α value of the acidic groups for PE-Glyc100 was found at 5.8 and increased to 6.4 for PE-Glyc50 and 6.5 for PE-Glyc80 (Figure 5 and Table 1). These values are again higher than the p*K*α = 3.8 of thioglycolic acid, due to the polyelectrolyte effect and the hydrophobicity of the polymer, discussed above, and increase even further for PE-Glyc50 and PE-Glyc80 bearing hydrophobic alkene side groups. It should also be noted that the effective p*K*α values for all the PE-GlycXX polyesters were lower compared to the respective values found for the PE-PropXX analogues, a difference that becomes more notable for the 100% modified polyesters, and is attributed to the shorter alkyl chain length of the polymer side groups, glycolic vs. propionic, which resulted in a decrease of the hydrophobicity of the polymer and facilitated the ionization of the acidic moieties. Overall, the p*K*α values of the synthesized polyesters varied in the range from 5.8 to 6.9, by appropriately adjusting the hydrophobicity/hydrophilicity of the polymer side groups, allowing the design of novel biodegradable and pH-sensitive polymers with the desired p*K*α value.

Besides the net charge along the polymer chains, the protonation/deprotonation process of the pendant carboxylic acid groups alters the polymer solubility in water. The pH-responsive behavior of the carboxylic acid functionalized polyesters was investigated by turbidimetry measurements. The transmittance of dilute aqueous polymer solutions at λ = 650 nm was measured as a function of the solution pH at room temperature (Figure 5). The pH at which the polymer solution becomes turbid is known as the transition pH and the respective values obtained for the functional polyesters prepared in this study are shown in Table 1.

PE-Prop50, exhibited a sharp transition in its transmittance at pH = 8.1, indicating that the polymer is soluble at high pH values and becomes hydrophobic below this pH. This transition is shifted to pH = 7.2 for PE-Prop80 and pH = 6.7 for PE-Prop100, suggesting that the copolymers become more hydrophilic as the fraction of the carboxylic acid side groups increases, as expected. Considering the degree of ionization as a function of the solution pH (Figure 4), it is deduced that PE-Prop50 is soluble in water only when 93% of the acidic side groups are ionized and becomes insoluble when the degree of ionization decreases. The degree of ionization values at the transition pH for PE-Prop80 and PE-Prop100 were found at ~67% and ~51%, respectively, indicating that the degree of ionization required to solubilize the polymer decreases with the fraction of carboxylic acid side groups increases. Interestingly, the above degrees of ionization at the transition pH correspond to ~50 ionized monomer repeat units in all cases, suggesting that despite the increase in the fraction of carboxylic acid pendant groups on the polymer chains, the number of ionized carboxylic acid units required to solubilize the polymers in water is almost constant.

A similar pH-responsive behavior was found for the copolymers functionalized with thioglycolic acid. PE-Glyc50 exhibited a phase transition at pH = 6.9, which corresponds to a 71% degree of ionization, compared to PE-Glyc80 at pH = 6.4 (degree of ionization 44%) and PE-Glyc100 at pH = 5.6 (degree of ionization 37%). Again, the transition pH decreases with the degree of modification of the alkene side groups, suggesting an increase in the hydrophilicity of the copolymer as the fraction of carboxylic acid side groups increases. Moreover, the degree of ionization at the transition pH decreases for PE-Glyc50, PE-Glyc80, and PE-Glyc100; however, the number of ionized monomer repeat units required to solubilize the polymers is again constant at ~36 units. It is noted that a comparison of the PE-GlycXX and PE-PropXX copolymers of the same degree of modification of the alkene side groups, shows that the transition pH and degree of ionization at the transition pH is lower for the former copolymers, indicating their higher hydrophilicity due to the shorter alkyl chain length of the functional pendant groups.

**Figure 6:**
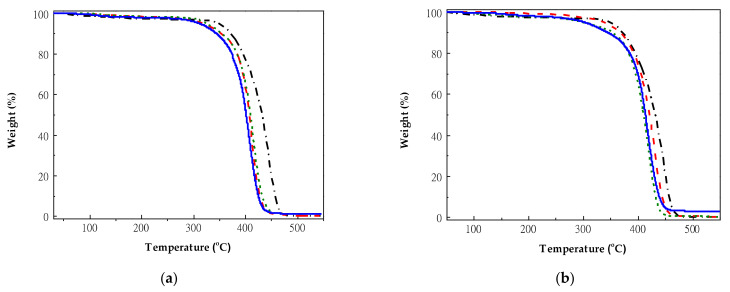
TGA curves of (**a**) P(TPAE‒*alt*‒AD) (black dot-dashed line), PE‒Prop50 (red dashed line), PE‒Prop80 (olive short dashed line), and PE‒Prop100 (blue solid line); (**b**) P(TPAE‒*alt*‒AD) (black dot-dashed line), PE‒Glyc50 (red dashed line), PE‒Glyc80 (olive short dashed line), and PE‒Glyc100 (blue solid line).

Next, thermogravimetric analysis was employed to study the thermal stability of the functional polyesters. As seen in the TGA curves (Figure 6), the degradation temperature of the acid functional polyesters is slightly lower compared to that of the alkene functional precursor polymer, which is attributed to the presence of the carboxylic acid groups that can undergo condensation and decarboxylation upon thermal heating. However, the fraction of the carboxylic acid side groups does not significantly affect the thermal stability of the polymers (Table 2). Overall, it can be concluded that all polymers are stable up to ~250 °C and can be safely heated up to this temperature without any decay.

### 3.3. Deposition of Thin Polyester Films on Glass Substrates

The synthesized functional polyesters combining carboxylic acid and alkene side groups are attractive materials for the preparation of pH-responsive polyester-based cross-linked polymers and films upon chemically linking their vinyl pendant groups. The biological properties of the synthesized functional polyesters were investigated using thin polymer films deposited on glass coverslips. For the preparation of the thin films, first the coverslips were modified with MAPTMS, to bear polymerizable methacrylate groups, which were next utilized to chemically link the film on the substrate by reacting them with the vinyl side groups present along the polyester backbone, and thus enhancing the adhesion and stability of the films. Subsequently, a 10 *w*/*v*% solution of each functional polyester in DMF, containing DMPA as the photoinitiator, was spin-coated onto the pre-silanized glass substrates. Following irradiation of the films at 365 nm for 90 min, to induce the cross-linking of the polymer and its chemical attachment on the substrates, stable polyester films were obtained (Scheme S1). PE-Prop100 and PE-Glyc100 were not spin coated onto the substrates, as these polymers do not possess any vinyl side-groups, and therefore, could not form stable cross-linked films strongly bound onto the substrates. The thickness of the dry cross-linked films was measured by profilometry and was found between 100–200 nm for all copolymer samples.

The hydrophilicity of a material surface is an important characteristic that strongly affects its biological properties, including cell adhesion and proliferation, among others. Therefore, the wettability of the cross-linked functional polyester films was investigated by static water contact angle (WCA) measurements (see Supplementary Materials, Figure S3) to ascertain the influence of the degree of modification of the alkene side-groups of the polymer, to carboxylic acid moieties, on the hydrophilicity of the polymer surface. Table 2 shows the WCA values obtained for the polyester films. The wettability was found similar for all films with a WCA of ~85°, which is also similar to the WCA of the alkene functional precursor polyester, except for the PE-Glyc80 film, which was found more hydrophilic with a WCA of 75°. These results suggest that the modification of the polymer to bear carboxylic acid side groups alone, does not enhance significantly the wettability of the films [22]. Polymers modified with the longer alkyl chain length mercaptocarboxylic acid, or at lower degrees of modification with the shorter alkyl chain length mercaptocarboxylic acid, remain barely hydrophilic, and a combination of both a shorter alkyl chain side group and a higher fraction of ionizable carboxylic acid pendant moieties are required to enhance the hydrophilic nature of the polymer surface.

### 3.4. Adhesion and Morphology of Fibroblasts Cultured on the Polyester Films

**Figure 7:**
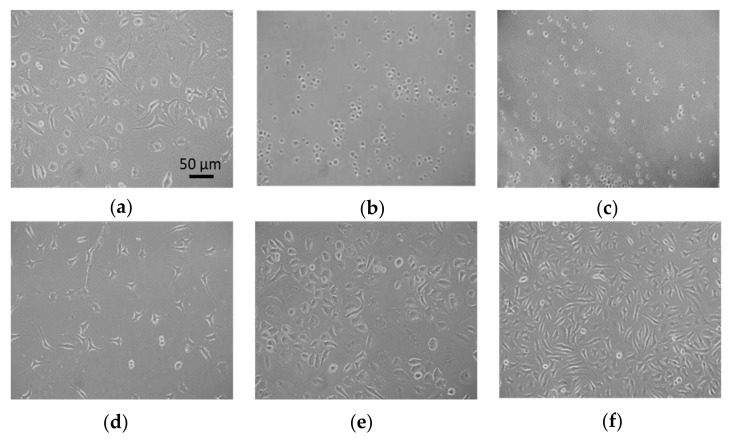
Optical microscopy images depicting the adhesion and morphology of L929 fibroblasts after 4 days in culture on the (**a**) TCPS control, (**b**) P(PTPAE‒*alt*‒AD), (**c**) PE‒Prop50, (**d**) PE‒Glyc50, (**e**) PE‒Prop80, and (**f**) PE‒Glyc80 films. Scale bar represents 50 μm and is the same in all images.

Inverse optical microscopy was used to assess the adhesion and morphology of L929 fibroblasts on the surface of the cross-linked polyester films and compare it to the TCPS control (Figure 7). All images were captured after 4 days in culture. The precursor polyester film (PTPAE-*alt*-AD) (Figure 7b) and the PE-Prop50 surface (Figure 7c) presented very low cell adhesion, with the few cells being visibly stressed and round, suggesting that these materials do not promote cell adhesion, viability, and proliferation. On the other hand, the PE-Glyc50 film (Figure 7d) showed a good cell adhesion, however, the survivability of the cells was moderate since the number of living cells on the film was significantly lower than that of the TCPS control (Figure 7a). Finally, the PE-Prop80 and PE-Glyc80 surfaces presented the best response with regards to cell adhesion, with the PE-Prop80 film showing similar adhesion to the TCPS control both in terms of cell morphology and number (Figure 7e), whereas, the PE-Glyc80 surface demonstrated an excellent cell attachment and the highest proliferation on day 4 compared to all other surfaces, including the TCPS control, visualized as a dense cell population with extended pseudopodia formed by all cells (Figure 7f).

Cell adhesion is a process that has been reported to play a significant role in the tissue integration of a biomaterial, as the biocompatibility of the biomaterials is closely related to the cellular behavior in contact with the material [28]. Therefore, cell attachment and spreading determine the initial cell-biomaterial interactions that will further affect the proliferative and differentiation potential of cells in contact with the implant. The surface properties of a biomaterial, including its chemistry, topography, and surface energy are essential for cell adhesion. Moreover, a biomaterial should be bioactive and capable of interacting with the surrounding cells, thus regulating specific cellular functions and guiding tissue development, while its degradation should match the tissue formation rate [29]. The evaluation of the attachment of several cell types on innovative polymeric biomaterials surfaces and structures has been the focus of our research in recent years [1,3,30,31]. Cell adhesion on biomaterial surfaces, together with other relevant cellular responses and specific functions, have been proved to be useful tools for the evaluation of the suitability of biomaterials to perform properly when they interact with cells and tissues in vitro and in vivo. The term biocompatibility is a complex characteristic of a system reflecting the successful outcome of an implantation in a clinical application, although its definition as “the ability of a material to perform with an appropriate host response in a specific application” has been widely used for more than 30 years [32].

### 3.5. Evaluation of the Cell Viability and Proliferation on Polyester Films

**Figure 8:**
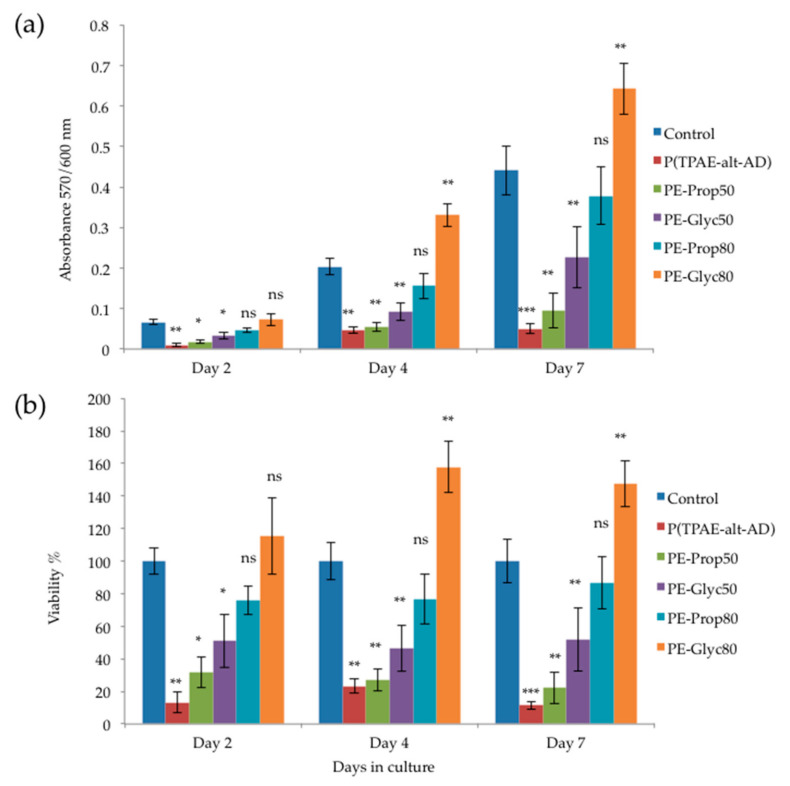
Cell viability and proliferation showing the absorbance at 570/600 nm (**a**) and the % viability (**b**) compared to the TCPS control on the functional polyester films on day 2, 4 and 7. A standard pattern in cell viability is present on day 2, which is also similar in days 4 and 7, except for the PE-Glyc80, which turns from statistically not significant, compared to the TCPS control on day 2, to statistically significant on days 4 and 7, clearly exceeding the TCPS control. Data are expressed as mean ± standard deviation. Asterisks denote statistical significance in viability for the functional polyesters compared to the TCPS control surface at each experimental time point (*: *p* < 0.05, **: *p* < 0.01, ***: *p* < 0.001, ns: not significant).

The cell viability and proliferation on the polyester surfaces was assessed by means of the PrestoBlue^TM^ cell viability assay on days 2, 4, and 7, and the results are presented as absorbance values at 570/600 nm (Figure 8a) and as % viability compared to the TCPS control (Figure 8b). On day 2, PE-Prop80 and PE-Glyc80 indicated the highest cell viability among the examined polymers, very similar to the TCPS control, with the PE-Glyc80 polyester slightly exceeding the TCPS control without statistical significance. The P(TPAE-*alt*-AD), PE-Prop50, and PE-Glyc50 polyesters presented significantly lower (*p* < 0.05) cell viability when compared to the TCPS control. On day 4, the cell viability follows the same trend for all polymer surfaces as day 2, with PE-Glyc80 demonstrating a statistically significant increase compared to the TCPS control (*p* < 0.01). Finally, on day 7, the viability indicates a similar pattern as that on day 4. At all experimental time points, it was found that PE-Glyc80 and PE-Prop80 favored the cell viability, compared to the respective 50% modified polyesters, PE-Glyc50 and PE-Prop50, with the PE-Prop80 reaching at least 80% and the PE-Glyc80 at least 128% of the viability on the TCPS control surface. These results demonstrate that the highest cell viability was observed for the polyester with the shorter alkyl chain length and the higher fraction of carboxylic acid groups, PE-Glyc80 with a WCA of 75°. Similar cell viability results on osteoblastic cell proliferation, which increase up to 7 days in culture, were described in a previous report on chitosan-*graft*-PCL (CS-*g*-PCL) copolymer films with WCA of 67° [1], as well as on CS-*g*-PLLA films with a WCA of 75° [3]. In addition, the CS-*g*-PCL films promoted the viability and proliferation of Wharton jelly mesenchymal stem cells [30], rendering these copolymers promising candidates for both hard and soft tissue engineering applications. To this direction, the immunomodulation of biomaterials is crucial, as their chemical composition can affect the polarization of macrophages and the outcome of the implantation. The differentiation of macrophages cultured on CS-*g*-PCL films has been reported to favor their transition from M1 to M2 phase, and thus, their anti-inflammatory activity, by increasing the content of the hydrophilic chitosan from 50% to 78% *w*/*v* [33]. Overall, these data underline the importance of the surface properties on the cellular viability, proliferative, and immunomodulatory potential. Notably, the WCA values of 65–75° in a biological context are rather to be seen as hydrophobic, as the transition from hydration repulsion to hydrophobic attraction occurs at a contact angle of θ ≈ 60°, which is referred to as the Berg limit [34,35]. However, as discussed in Section 3.4 above, other biomaterials surface properties including their chemistry, topography, and surface energy are essential for the attachment, migration, proliferation and differentiation of cells.

## 4. Conclusions

In summary, main chain polyesters bearing alkene side groups were prepared via a condensation copolymerization of a diol with a diacid chloride comonomer. The main chain degradable polymers were further functionalized to bear carboxylic acid pendant groups via photo-induced thiol-ene chemistry. Two different mercaptocarboxylic acids, 3-mercaptopropionic acid and thioglycolic acid, were employed to functionalize the precursor polymer with the pH-responsive weak acid functionalities. The degree of modification was varied by tuning the irradiation time between 40 s to 5 min. Three different functional polyesters were prepared in each case with ~50, ~80 and 100% carboxylic acid side groups. The functional polyesters exhibited a pH-responsive behavior and solubility in aqueous media as a function of the degree of ionization of the pendant acidic moieties. This resulted in polymers with tunable p*Κ*_α_ values and tunable solubility ranging from hydrophobic and water insoluble to hydrophilic and water soluble as a function of the solution pH. Cross-linked thin polyester films were prepared by the deposition of the polymers on pre-silanized glass substrates followed by light irradiation to chemically link the pendant alkene functionalities. The films were marginally hydrophilic, with water contact at angles of ~85°, except for the polyester modified with 80% thioglycolic acid, PE-Glyc80, which possessed the shorter alkyl chain length and showed a higher hydrophilicity with a water with a contact angle of 75°. Cell viability and proliferation studies on the cross-linked polyester films showed a superior performance of the PE-Glyc80 surface, which promoted the adhesion and growth of L929 fibroblasts to a greater extent compared to the TCPS control surface, verifying an excellent cell behavior on the polymer. Further studies are underway, to investigate the use of this polymer in 3D cell cultures as a functional, bioactive material in tissue engineering applications.

## Figures and Tables

**Table 1:** Functional polyesters and their characterization data.

Functional Polyesters	Degree of Modification (%) ^1^	p*K*α ^2^	Transition pH ^3^
P(TPAE-*alt*-AD)	-	-	-
PE-Prop50	49	6.9	8.1
PE-Prop80	83	6.9	7.2
PE-Prop100	100	6.6	6.7
PE-Glyc50	46	6.4	6.9
PE-Glyc80	83	6.5	6.4
PE-Glyc100	100	5.8	5.6

^1^ Determined by ^1^H NMR spectroscopy. ^2^ Determined by potentiometric titrations. ^3^ Determined by optical transmittance measurements.

**Table 2:** Degradation temperature of the functional polyesters and WCAs on thin polymer films.

Sample	Degradation Temperature ^1^ (°C)	Water Contact Angle (°)
P(TPAE-*alt*-AD)	447	83 ± 1
PE-Prop50	410	86 ± 1
PE-Prop80	414	84 ± 1
PE-Prop100	406	-
PE-Glyc50	428	83 ± 1
PE-Glyc80	418	75 ± 1
PE-Glyc100	419	-

^1^ Determined by TGA.

## Data Availability

The data presented in this study are available on request from the corresponding author.

## Supplementary Materials

The following are available online at https://www.mdpi.com/article/10.3390/polym13101636/s1, Figure S1: Potentiometric titration curves of the PE-PropXX copolymers, Figure S2: Potentiometric titration curves of the PE-GlycXX copolymers, Scheme S1: Procedure for the thin film preparation, Figure S3: Static water contact angles on the cross-linked thin polymer films.
