# Supplementary material for: Responsive Polyesters with Alkene and Carboxylic Acid Side-Groups for Tissue Engineering Applications
Source: Polymers (Basel). 2021 May 18;13(10):1636. doi: 10.3390/polym13101636 (PMC8158382; doi:10.3390/polym13101636)
Supplement: Supplementary file 1 [file polymers-13-01636-s001.zip › polymers-1216693-supplementary.pdf]

polymers

# Supplementary Materials

# Responsive Polyesters with Alkene and Carboxylic Acid Side-Groups for Tissue Engineering Applications

**Stella Afroditi Mountaki [1,2], Maria Kaliva [1,3], Konstantinos Loukelis [1,3], Maria Chatzinikolaidou [1,3] and Maria Vamvakaki [1,3,*]**

[1] Institute of Electronic Structure and Laser, Foundation for Research and Technology-Hellas, Vassilika Vouton, 700 13 Heraklion, Greece; smountaki@gmail.com (S.A.M.); kalivm@iesl.forth.gr (M.K.); loukelisk@yahoo.com (K.L.); mchatzin@materials.uoc.gr (M.C.)
[2] Department of Chemistry, University of Crete, Vassilika Vouton, 700 13 Heraklion, Greece
[3] Department of Materials Science and Technology, University of Crete, Vassilika Vouton, 700 13 Heraklion, Greece
* Correspondence: vamvakak@materials.uoc.gr

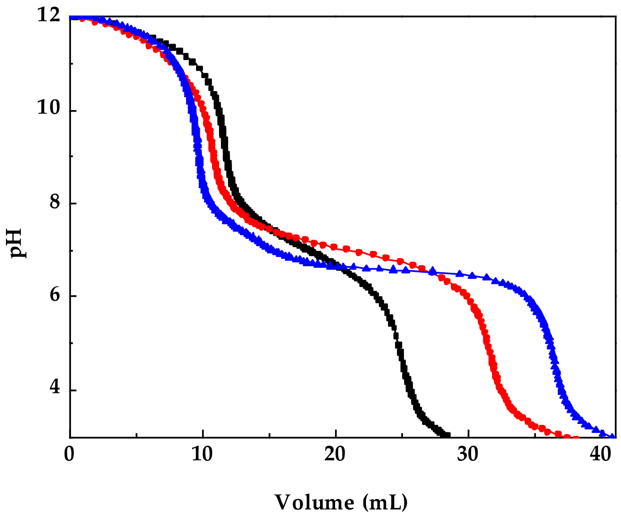


**Figure S1.** Potentiometric titration curves for a 10 mg mL$^{-1}$ solution of PE-Prop50 (■), PE-Prop80 (●) and PE-Prop100 (▲).

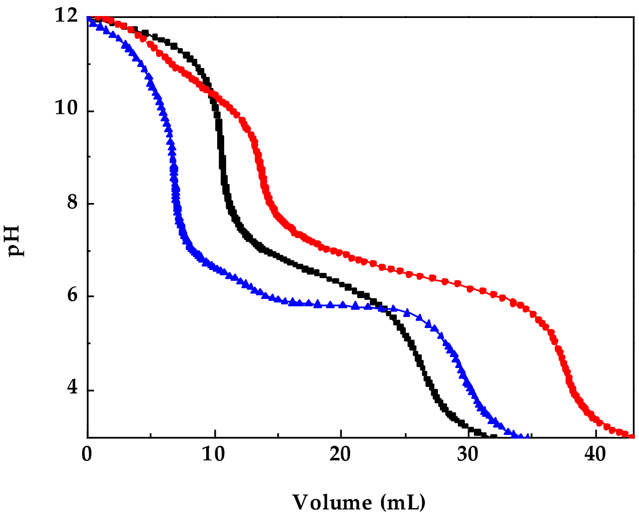


**Figure S2.** Potentiometric titration curves for a 10 mg mL$^{-1}$ solution of PE-Glyc50 (■), PE-Glyc80 (●) and PE-Glyc100 (▲).

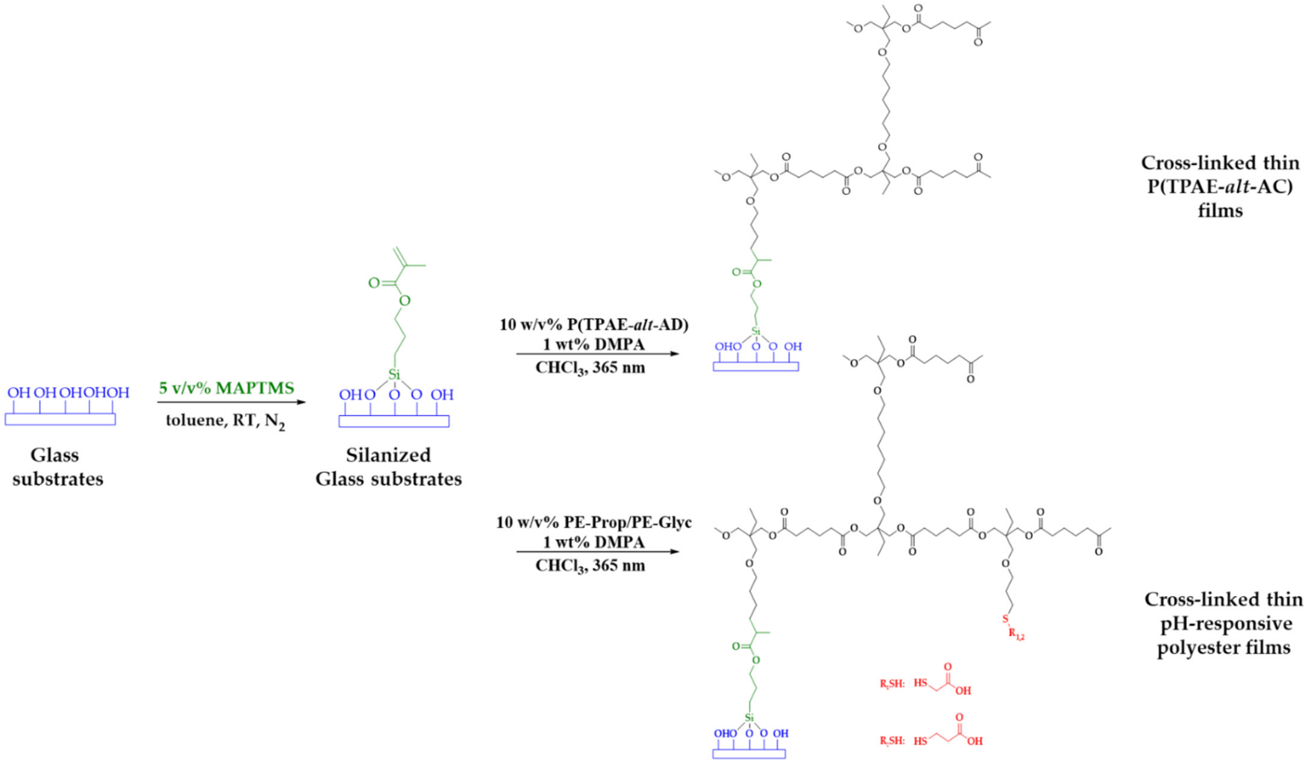


**Scheme S1.** Schematic representation of the synthetic procedure followed for the preparation of the polyester films.

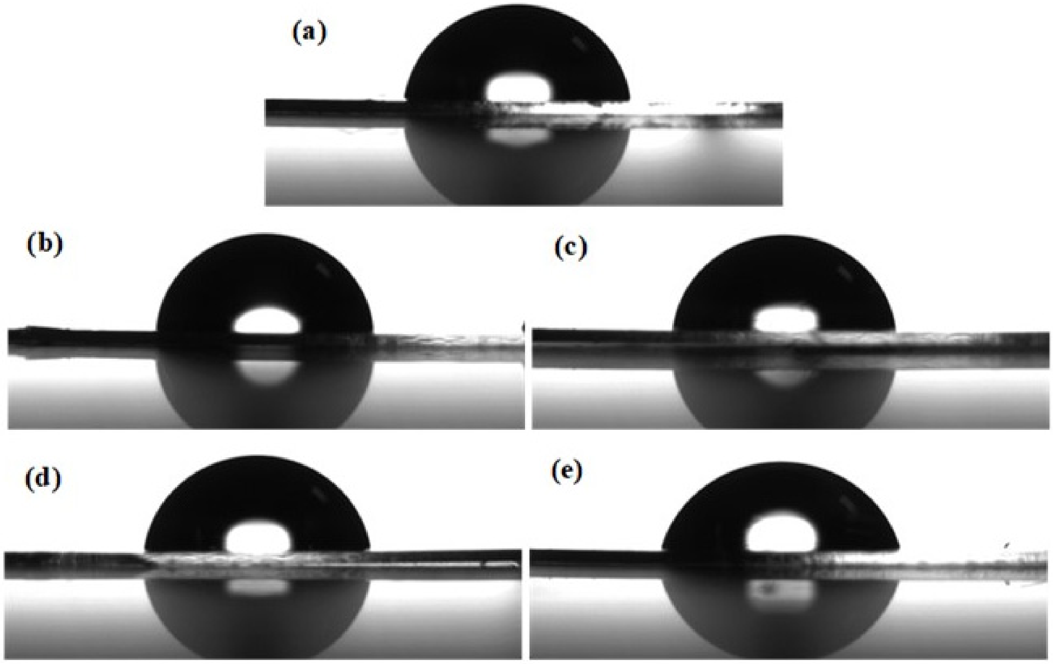


**Figure S3.** Static water contact angles on cross-linked thin films of (a) P(TPAE-*alt*-AD), (b) PE-Prop50, (c) PE-Prop80, (d) PE-Glyc50 and (e) PE-Glyc80.
